# Machine learning-driven identification of exosome- related biomarkers in head and neck squamous cell carcinoma

**DOI:** 10.3389/fimmu.2025.1590331

**Published:** 2025-05-22

**Authors:** Yaodong He, Yun Li, Jiaqi Tang, Yan Wang, Zhenyan Zhao, Rong Liu, Zihui Yang, Huan Li, Jianhua Wei

**Affiliations:** State Key Laboratory of Oral and Maxillofacial Reconstruction and Regeneration, National Clinical Research Center for Oral Diseases, Shaanxi Clinical Research Center for Oral Diseases, Department of Oral and Maxillofacial Surgery, School of Stomatology, The Fourth Military Medical University, Xi’an, China

**Keywords:** head and neck squamous cell carcinoma, exosome biomarkers, machine learning, immune microenvironment, therapeutic target discovery

## Abstract

**Background:**

Head and neck squamous cell carcinoma (HNSCC) is a common cancer associated with elevated mortality rates. Exosomes, diminutive extracellular vesicles, significantly contribute to tumour development, immunological evasion, and treatment resistance. Identifying exosome-associated biomarkers in HNSCC may improve early diagnosis, treatment targeting, and patient classification.

**Methods:**

We acquired four publically accessible HNSCC gene expression datasets from the Gene Expression Omnibus (GEO) database and mitigated batch effects utilising the ComBat technique. Differential expression analysis and exosome-related gene screening found a collection of markedly exosome-associated differentially expressed genes (ERDEGs). Subsequently, 10 key exosome-related genes were further screened by combining three machine learning methods, LASSO regression, SVM-RFE and RF, and a clinical prediction model was constructed. Furthermore, we thoroughly investigated the biological roles of these genes in HNSCC and their prospective treatment implications via functional enrichment analysis, immune microenvironment assessment, and molecular docking confirmation.

**Results:**

The study indicated that 10 pivotal exosome-related genes identified by the machine learning method had considerable differential expression in HNSCC. Clinical prediction models developed from these genes have shown high accuracy in prognostic evaluations of HNSCC patients. Analysis of the immunological microenvironment indicated varying immune cell infiltration in HNSCC, and the association with ERDEGs proposed a potential mechanism for immune evasion. Molecular docking validation indicated novel small molecule medicines targeting these genes, establishing a theoretical foundation for pharmacological therapy in HNSCC.

**Conclusion:**

This research identifies new exosome-related indicators for HNSCC through machine learning methodologies. The suggested biomarkers, particularly ANGPTL1, exhibit significant promise for diagnostic and prognostic uses. The investigation of the immunological microenvironment yields insights into immune modulation in HNSCC, presenting novel avenues for therapeutic targeting.

## Introduction

Head and Neck Squamous Cell Carcinoma (HNSCC) is among the most prevalent malignant neoplasms of the head and neck, with significant morbidity and mortality rates globally (1, 2). Notwithstanding advancements in the diagnosis and treatment of HNSCC in recent years, the prognosis for patients, particularly those in advanced stages, remains unfavourable, characterised by a low five-year survival rate (3, 4). Consequently, a thorough investigation of the molecular pathways of HNSCC, together with identifying novel biomarkers and prospective therapeutic targets, is crucial for enhancing the clinical management of patients.

In recent years, exosomes, as significant extracellular vesicles, have garnered considerable attention in tumour biology research. An exosome is a nanoscale vesicle released by cells, abundant in biomolecules, including proteins, RNA, DNA, and lipids, which can modulate the tumour microenvironment via intercellular communication and is pivotal in carcinogenesis, progression, metastasis, and medication resistance (5–7). Research indicates that exosomes play a role in tumour cell signalling and affect tumour immune evasion by modulating immune cell activity (8). Moreover, exosomes’ particular molecular constituents (e.g., miRNAs, lncRNAs, and proteins) have demonstrated significant diagnostic and prognostic significance across various malignancies (9, 10). The precise functions of exosome-related genes in HNSCC and their potential as biomarkers have not been comprehensively examined.

Concurrently, machine learning (ML) is progressively employed as a potent data analysis instrument in the biomedical sector. Machine learning can extract essential elements from extensive datasets using algorithms, develop prediction models, and offer accurate disease diagnosis, classification, and therapy assistance (11–13). In tumour research, machine learning has been effectively utilised for analysing gene expression data, biomarker screening, and developing clinical prognostic models (14, 15). The integration of machine learning and exosome-associated gene study in HNSCC remains nascent, and its potential has yet to be thoroughly investigated.

This study systematically identified exosomal biomarkers in HNSCC by integrating multi-omics data and machine learning. We explored their roles in the tumour immune microenvironment and drug discovery. Four HNSCC gene expression datasets were obtained from the Gene Expression Omnibus (GEO) database, with batch effects mitigated via the ComBat technique to ensure uniformity. Through differential expression analysis, exosome-associated gene screening, and functional enrichment, we identified highly differentiated exosome-related genes (ERDEGs). Three machine learning approaches—Least absolute shrinkage and selection operator (LASSO) regression, Support Vector Machine Recursive Feature Elimination (SVM-RFE), and Random Forest (RF)—were integrated to pinpoint 10 core exosome-related genes, enabling the development of a clinical prediction model. Additionally, we analysed associations between these genes and the immunological microenvironment, while screening potential small-molecule drugs, thereby providing a theoretical basis for future translational research.

## Materials and methods

### Data acquisition and preprocessing

Four HNSCC gene expression datasets—GSE25099 (57 tumours vs. 22 normals from Taiwan, Affymetrix), GSE30784 (167 tumours vs. 45 normals from US, Affymetrix), GSE37991 (40 tumour-normal pairs from Taiwan, Illumina), and GSE127165 (57 laryngeal SCC-normal pairs from China, Illumina)—were retrieved from GEO and harmonised using ComBat batch correction (sva v3.46.0) to preserve biological variance while eliminating platform-specific technical artefacts. Raw microarray data underwent rigorous preprocessing: RMA background correction with quantile normalisation, log2 transformation, and filtering of genes expressed (CPM > 1) in ≥ 50% samples. Quality control retained samples with median intensity > 2 SDs above cohort mean and > 85% detection rate, alongside genes exhibiting > 0.2 coefficient of variation (CV). Missing values were imputed via k-nearest neighbours (k = 15), with batch effect removal efficacy confirmed through principal component analysis (PCA) clustering patterns and interquartile range consistency in boxplots.

### Differential expression analysis

Differentially expressed genes (DEGs) were identified using the Limma pipeline, defined by statistical significance (p < 0.05) and absolute log2 fold change (|log2FC|) > 1. Results were visualised through a heatmap (pheatmap R package) displaying hierarchical clustering of top DEGs across samples, and a volcano plot (ggplot2 R package) contrasting log2FC against—log10 (p-value), with significant DEGs highlighted.

### Exosome-related gene screening

Exosome-related genes were extracted from the GeneCards database (**Supplementary Table 1**). Genes linked to exosomes were found using the search phrase “exosome” and filtered according to a relevance score > 2 to guarantee high-confidence relationships. The list of DEGs derived from the Limma pipeline was cross-referenced with the curated exosome-related gene list. The Venn diagram was created utilising the VennDiagram R tool, visually illustrating the intersection between the two gene sets. Genes located in the intersection were identified as exosome-related differentially expressed genes (ERDEGs).

### Functional enrichment profiling

Gene Ontology (GO) and Kyoto Encyclopaedia of Genes and Genomes (KEGG) pathway enrichment studies were conducted utilising the clusterProfiler R package to investigate the biological activities and pathways related to the ERDEGs. The enrichment analysis was conducted using a significance threshold of adjusted p-value < 0.05, and the findings were illustrated using bar graphs and dot plots. Gene Set Enrichment Analysis (GSEA) was performed to further examine the functional characteristics of the ERDEGs at the gene set level. The Hallmark gene sets from the Molecular Signatures Database (MSigDB) served as the reference gene sets. GSEA was conducted utilising the fgsea R package, and enrichment scores were computed to ascertain gene sets significantly enriched in the ERDEGs. The results were illustrated by enrichment plots, with the foremost enriched gene sets given according to their normalised enrichment score (NES) and a false discovery rate (FDR) < 0.25.

### Machine learning-based biomarker discovery

Three machine learning approaches were sequentially applied for feature selection: (1) LASSO regression (glmnet v4.1-6) performed dimensionality reduction via L1 regularisation, where the optimal λ value minimising prediction error was determined through 10-fold cross-validation, retaining genes with non-zero coefficients as candidate biomarkers; (2) SVM-RFE (e1071 v1.7-13) iteratively refined the feature subset by recursively eliminating lowest-weight features based on linear kernel SVM classifier performance until peak classification accuracy was achieved; (3) RF (randomForest v4.7-1.1) quantified feature importance via Gini impurity reduction across 500 decision trees, with final biomarker prioritisation based on descending importance scores, thereby establishing a robust multi-algorithm consensus for subsequent translational validation.

### Clinical predictive model construction

Receiver Operating Characteristic (ROC) curve analysis was performed to assess the efficacy of the clinical predictive model. The ROC curve was produced with the pROC R package, which computes the area under the curve (AUC) to assess the model’s discriminatory capacity. A nomogram was created with the Regression Modelling Strategies (RMS) R package to enhance the clinical implementation of the predictive model. The nomogram graphically illustrates the correlation between predictor variables and the anticipated outcome, enabling doctors to assess the likelihood of a specific clinical event for individual patients. The model’s coefficients were utilised to allocate point values to each predictor, and the cumulative points were correlated with the projected likelihood. Calibration curves were constructed to evaluate the concordance between expected and observed outcomes, confirming the nomogram’s reliability.

### Immune microenvironment characterisation

Single-sample Gene Set Enrichment Analysis (ssGSEA) was conducted utilising the LM22 signature matrix, which encompasses gene expression profiles of 22 immune cell types to delineate the immune cell composition inside the tumour microenvironment. The Gene Set Variation Analysis (GSVA) R software was utilised to compute enrichment scores for each immune cell type in individual samples. Box plots illustrated the findings to emphasise discrepancies in immune cell prevalence among samples. The relationship between ERDEGs and immune cell infiltration was assessed using Spearman correlation, followed by visualisation of the results with the pheatmap software.

### Drug sensitivity prediction

To forecast drug sensitivity based on the discovered ERDEGs, drug-gene connection data were sourced from the Drug Signatures Database (DSigDB). Drug enrichment analysis was conducted to find possible therapeutic agents that target ERDEGs. The fgsea R package was utilised for the study, wherein ERDEGs were evaluated for enrichment against the drug-gene sets derived from DSigDB. The enrichment scores were computed, and statistical significance was evaluated with an FDR < 0.25. The outcomes were prioritised according to the NES, and the most enriched pharmaceuticals were determined. The data were visualised through bar and enrichment plots, emphasising the most promising compounds for further examination.

### Molecular docking validation

The three-dimensional structures of the target proteins were obtained from the AlphaFold Protein Structure Database to confirm the interactions between projected drug candidates and their target proteins. The three-dimensional structures of small-molecule compounds found by drug sensitivity prediction were obtained from the PubChem database. Molecular docking simulations were performed utilising AutoDock Vina, a prevalent method for forecasting ligand-protein interactions. The target protein and small-molecule compounds were formatted in PDBQT, and a grid box was established to surround the putative binding site. Docking simulations used an exhaustiveness parameter of 8 to guarantee comprehensive sampling of the binding conformations. The highest-ranking postures’ binding affinities (measured in kcal/mol) were evaluated, and the findings were illustrated using PyMOL to investigate the molecular interactions.

### Regulatory network analysis

The starBase database examined the relationships between RNA-binding proteins (RBPs) and their target transcripts. The outcomes were refined according to high-confidence connections (e.g., corroborated by numerous CLIP-seq datasets), and the RBP-target gene network was illustrated using Cytoscape to emphasise critical regulatory linkages. The Transcriptional Regulatory Relationships Unravelled by Sentence-based Text Mining (TRRUST) database was utilised to deduce transcription factor (TF) regulatory interactions. The interactions between transcription factors and target genes were extracted to form a regulatory network. The network was depicted using Cytoscape, with nodes symbolising transcription factors and target genes and edges denoting regulatory interactions.

### Cell lines

Human Oral Keratinocytes (HOK) and human HNSCC cell lines, HN4, HN6, SCC9, and CAL27, were obtained from Wuhan Pricella Biotechnology Co. Ltd. DMEM medium was used for cultivation. The above medium was supplemented with 10% foetal bovine serum and 1% penicillin/streptomycin. All cell lines were cultured in a cell incubator at 37°C with 5% CO2 concentration.

### RNA extraction and quantitative real-time polymerase reaction

Total RNA was extracted using a silica-membrane column-based purification kit (Takara #9767), wherein the gDNA-Eraser column adsorbed genomic DNA while the RNA Pure column selectively bound RNA, yielding high-purity total RNA. Reverse transcription was performed with PrimeScript™ RT Reagent Kit (Takara #RR037A), followed by SYBR Green-based qPCR (Takara) on a Bio-Rad CFX96 system. Reactions were conducted in duplicate under two-step cycling: 95°C/30 sec denaturation, 40 cycles of 95°C/5 sec and 60°C/30 sec. GAPDH served as endogenous control, with relative gene expression quantified via 2−ΔΔCt method against normalised cycle threshold (Ct) values.

### Plasmids design and transfection

Firstly, the primers of angiopoietin like 1 (ANGPTL1) gene were designed by Anhui General Gene Technology Co., Ltd. and amplified by PCR, and the ends of cDNA were digested using XbaI and BamHI restriction endonucleases, and the pcDNA3.1(+) empty vector was digested in the same way; then, DNA ligase was utilised to ligate the amplified target fragment with the vector, and pcDNA3.1(+)-ANGPTL1 (containing the target vector for the ANGPTL1 gene) was obtained, and the ligated product was transformed into the receptor cells. The ANGPTL1 plasmid construction was successful after shaking the bacteria, coating the plate, selecting the positive clones, sequencing, amplifying the bacterial solution, and carrying out plasmid extraction and purification.

### Cell counting kit-8 proliferation assay

Transfected SCC9 and CAL27 cells were seeded in 96-well plates at a density of 2,000 cells per well (six replicates per group). Cell proliferation was assessed at 0, 24, 48, and 72 h. For CCK-8 assays, 10 μL of CCK-8 solution was added to each well containing 100 μL of culture medium. After incubation at 37°C for 2 h, absorbance at 450 nm was measured using a microplate reader to quantify proliferation differences between groups.

### Colony formation assay

Transfected SCC9 and CAL27 cells were seeded in 6-well plates (200 cells/well) and cultured under standard conditions. The medium was refreshed every 3 days for 1–2 weeks until visible colonies formed. Cells were then washed with PBS, fixed with 4% paraformaldehyde for 30 min, and stained with 0.1% crystal violet for 10 min. After rinsing to remove excess stain, plates were air-dried at room temperature. Colony numbers were quantified using ImageJ software.

### Wound healing assay

The transfected SCC9 and CAL27 cells were added to 6-well plates with 5×10^5^ cells per well, respectively. Incubate in the incubator overnight. Two parallel lines were drawn in the 6-well plate with a 100 μL pipette tip the next day. Wash the cells with PBS solution and add serum-free medium. Continue to incubate in the incubator, and observe and photo record under the inverted microscope at 0 h and 24 h.

### Transwell migration and invasion assays

Transfected SCC9 and CAL27 cells were resuspended at 1 × 10^5^ cells/mL. 100 μL cell suspension (10,000 cells/well) was seeded into Transwell inserts, with 600 μL medium containing 30% FBS added to the lower chamber. After 24 h incubation, inserts were fixed with 4% paraformaldehyde (10 min), stained with 0.1% crystal violet (15 min), and washed with PBS. Non-migrated cells on the upper membrane surface were removed by cotton swab. Migrated cells were imaged under a light microscope and quantified using ImageJ. For the invasion test, the protocol matched the migration assay except that transwell membranes were pre-coated with Matrigel (BD Biosciences; 1:8 dilution in serum-free medium) for 1 h at 37°C before cell seeding.

### Statistical analysis

Statistical analyses were performed using GraphPad 9.4.1. The Mann-Whitney U test was used to compare the means between the two groups, which are characterised by continuous measures that are not normally distributed. The t-test was used to analyse the comparison of means between the two groups, which are characterised by the need to conform to normally distributed measures. The chi-square test was used to analyse the difference between the two groups for count data.

## Results

### Identification of ERDEGs in HNSCC

**Figure 1:**
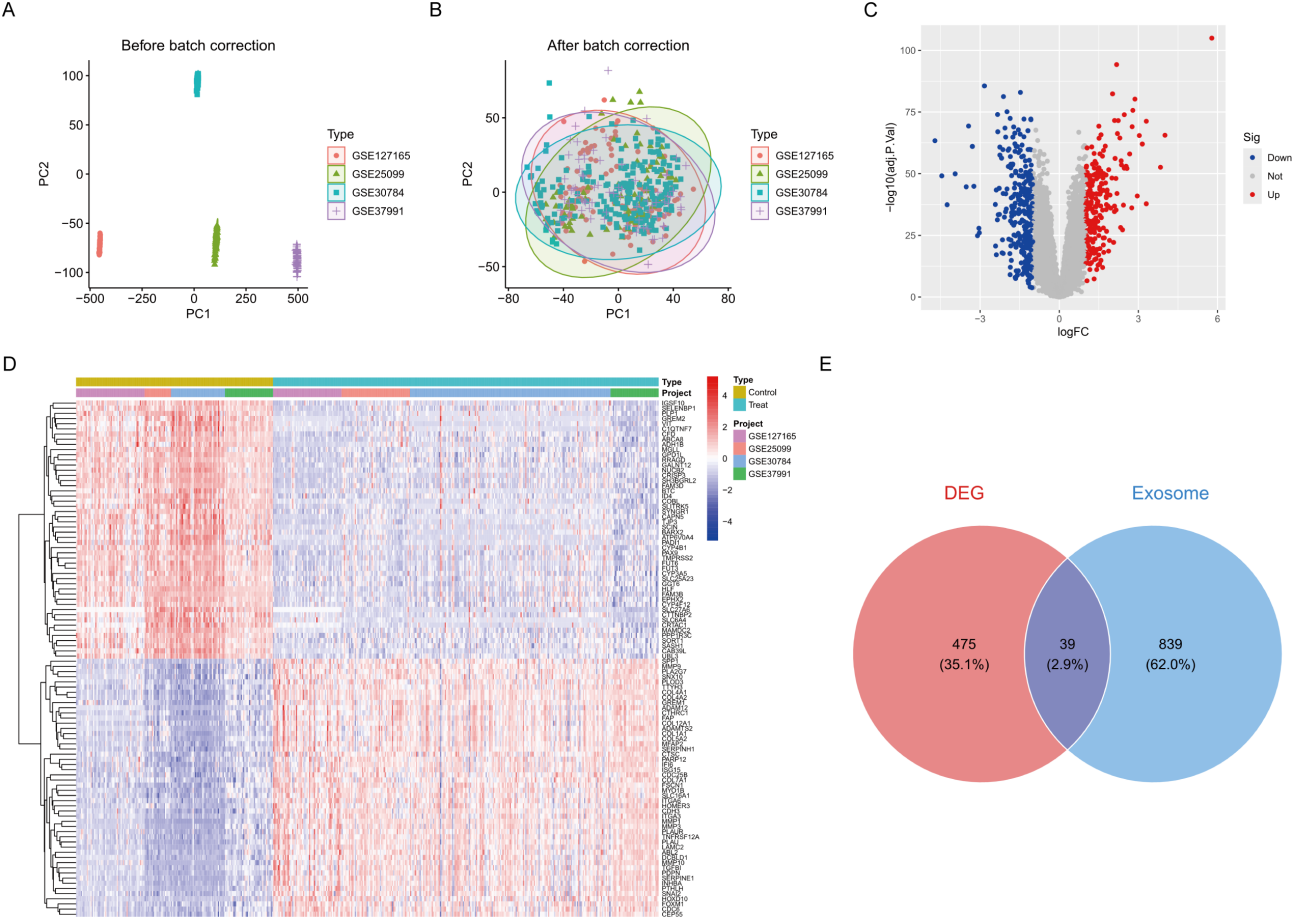
Differential expression analysis. **(A)** Pre-batch-corrected PCA plot. **(B)** Post-batch-corrected PCA plot. **(C)** Volcano plot of DEGs. **(D)** Heatmap of top 50 DEGs across cohorts. **(E)** Venn diagram of DEG-exosome gene intersection.

We developed a comprehensive analytical framework by integrating four separate HNSCC datasets (GSE25099, GSE30784, GSE37991, and GSE127165), which included 321 tumour samples and 164 normal tissue samples. PCA indicated substantial batch effects among cohorts before normalisation (**Figure 1A**, **Supplementary Figure 1B**). After ComBat batch correction, the variation in batch effects on gene expression distribution was substantially eliminated across all cohorts (**Figure 1B**, **Supplementary Figure 1B**). Utilising the limma program (p < 0.05, |log2FC| > 1), we found 514 consistently dysregulated genes across all datasets. The volcano plot identified 237 upregulated genes and 277 downregulated genes (**Figure 1C**). Hierarchical clustering of the top 50 differentially expressed genes distinctly separated tumour from normal tissues (**Figure 1D**). We curated 878 experimentally confirmed exosome-related genes from the GeneCards database (Relevance score > 2). The investigation of the intersection between DEGs and exosome genes identified 39 ERDEGs (**Figure 1E**).

### Functional enrichment analysis of ERDEGs

**Figure 2:**
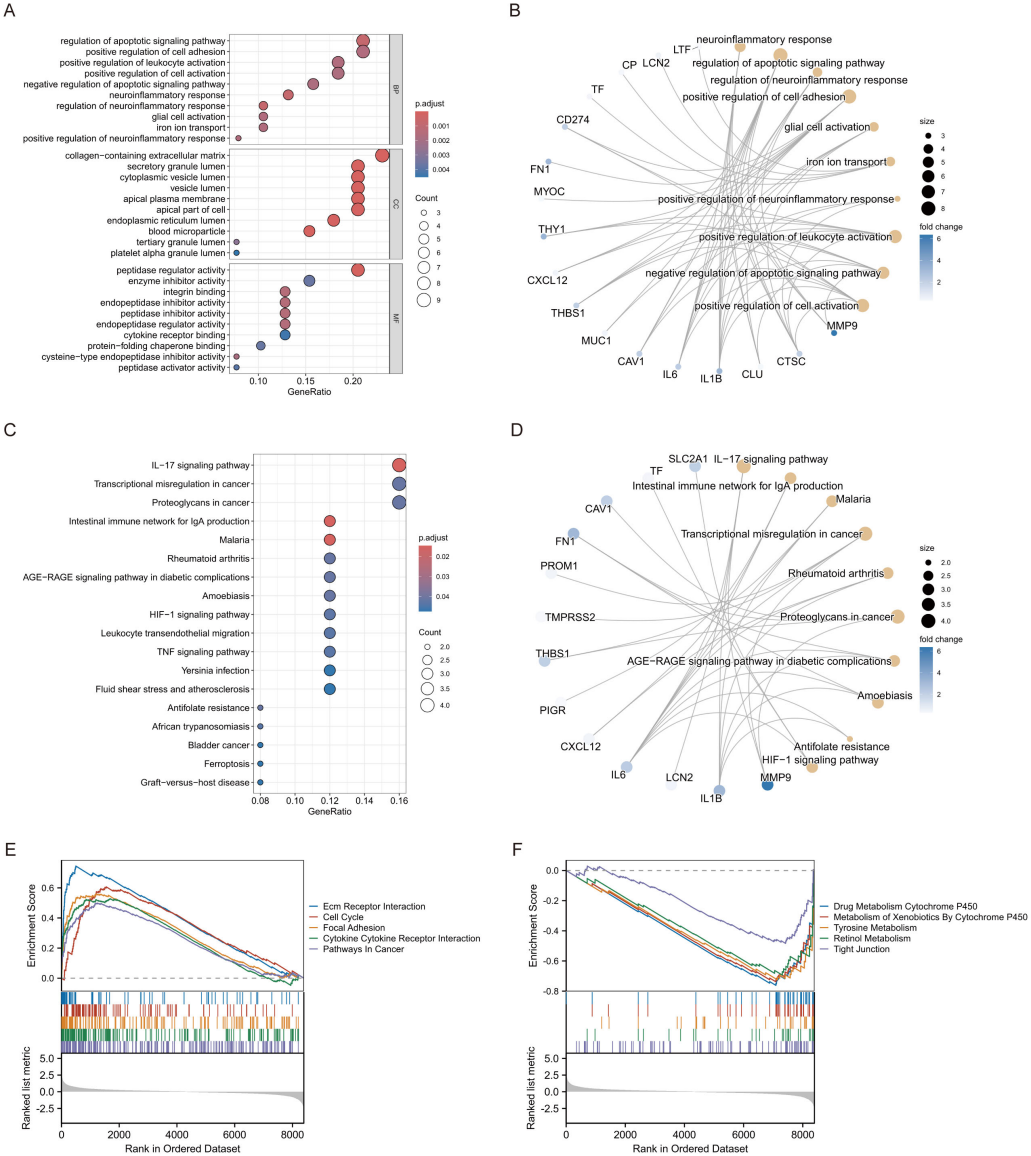
Enrichment analysis of ERDEGs. **(A)** GO enrichment analysis of ERDEGs. **(B)** Network diagram of ERDEGs with functional correlations. **(C)** KEGG enrichment analysis of ERDEGs. **(D)** Network diagram of ERDEGs related to pathway. **(E, F)** GSEA enrichment analysis of ERDEGs.

GO enrichment study identified numerous considerably enriched biological processes, cellular components, and molecular functions. The most significant biological processes encompassed the positive regulation of neuroinflammatory responses and the positive regulation of leukocyte activation, indicating the potential involvement of these genes in immunological responses and neuroinflammatory pathways. Enriching the vesicle lumen and secretory granule lumen indicates that these genes may participate in vesicular transit and secretion. The enrichment of cytokine receptor binding and protease inhibitory activity indicates a significant involvement of these genes in immunological signalling and protease regulation (**Figure 2A**). KEGG pathway analysis identified several pathways strongly linked to the genes, including fluid shear stress and atherosclerosis, graft-versus-host disease, and ferroptosis. Enriching the TNF signalling pathway and the rheumatoid arthritis pathway indicates that these genes may be pivotal in inflammatory responses and autoimmune disorders (**Figure 2C**). To enhance our comprehension of the association between genes, functions, and pathways, we further developed a gene-function network relationship map and a gene-pathway network relationship map. The gene-function network diagram illustrated the strong correlation between genes and essential functions, including immune response and neuroinflammation (**Figure 2B**). In contrast, the gene-pathway network diagram elucidated how these genes performed their biological roles by engaging in various significant signalling pathways (e.g., IL-17 signalling pathway, TNF signalling pathway, etc.) (**Figure 2D**). The network maps corroborated the aforementioned enrichment analysis findings and offered novel insights into the probable processes of genes in disease. Moreover, GSEA analysis corroborated the activation of numerous significant signalling pathways, including Cell Cycle and Cytokine Cytokine Receptor Interaction, which exhibited robust positive enrichment. The substantial enrichment of pathways, including ECM-receptor interaction and Cell Cycle, indicates their potential roles in cell adhesion and division, which may be linked to tissue remodelling and cancer progression (**Figure 2E**). Metabolic pathways, including Drug Metabolism Cytochrome P450, Metabolism of Xenobiotics by Cytochrome P450, and Tyrosine Metabolism, exhibited significant negative enrichment, indicating that these genes may be crucial in drug metabolism and the detoxification of exogenous compounds (**Figure 2F**).

### Machine learning-based biomarker discovery

**Figure 3:**
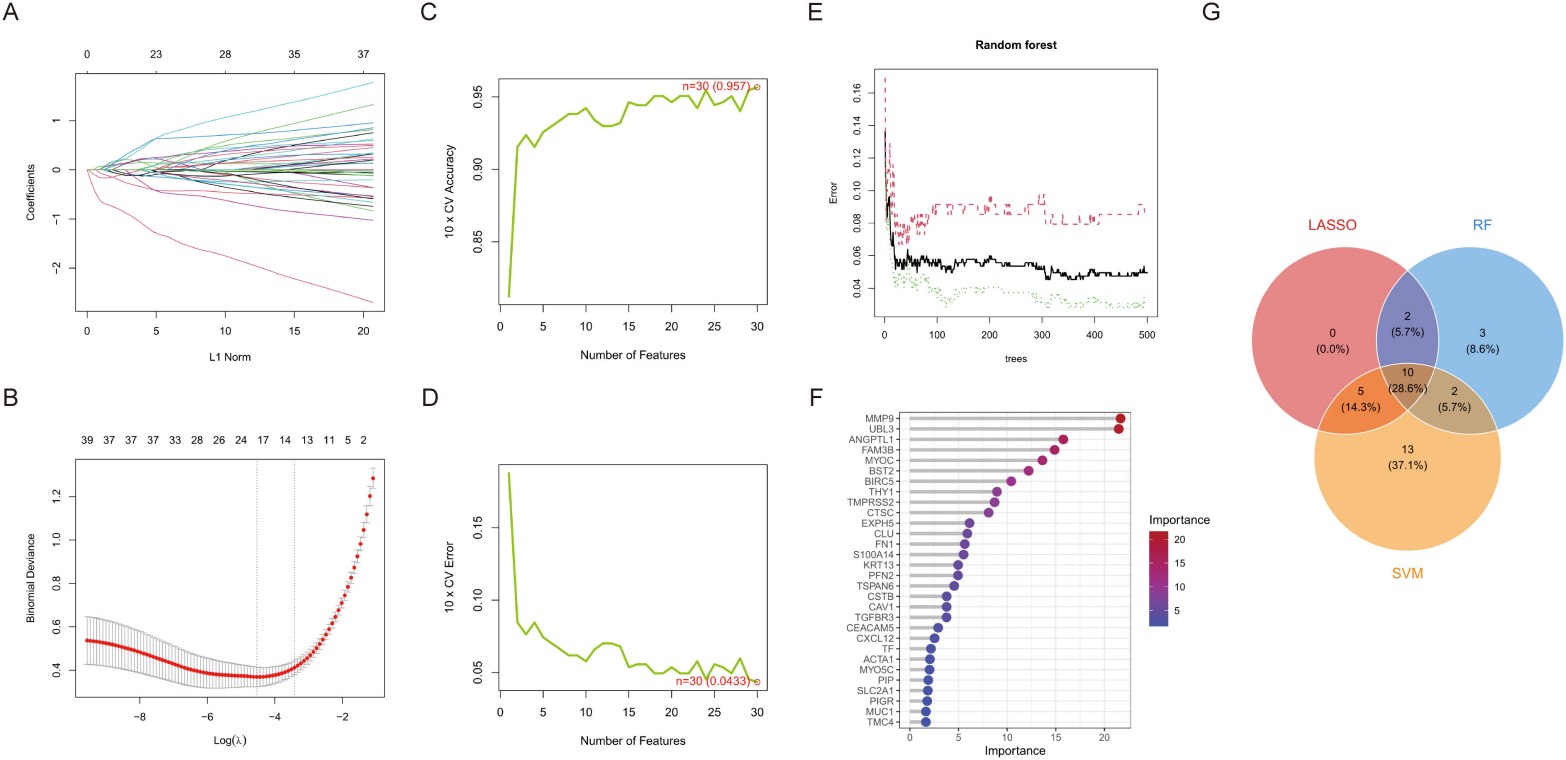
Machine learning screens for disease characterising genes. **(A)** Change in model bias under cross-validation. **(B)** LASSO regression coefficient L1 paradigm change. **(C)** Cross-validation accuracy and number of features change. **(D)** Cross-validation error and number of features change. **(E)** Plot of number of trees versus error rate in RF. **(F)** Ranking of importance of genetic variables in contributing to model prediction. **(G)** Venn diagram of LASSO, RF and SVM-RFE selected feature genes.

We conducted a one-way logistic regression analysis with a significance threshold of p < 0.05 to develop the HNSCC risk model, initially identifying 39 critical ERDEGs. This work employs three machine learning algorithms—LASSO, SVM-RFE, and RF—concurrently to improve the reliability of feature selection through comprehensive screening. LASSO regression effectively handles high-dimensional data by incorporating L1 regularisation, filtering out 17 essential ERDEGs while maintaining predictive efficacy. This method is particularly suited for datasets with many features, as it promotes sparsity in the model by selecting the most influential variables (**Figures 3A, B**). SVM-RFE iteratively eliminates less important features based on classifier accuracy, ultimately identifying 30 optimal candidate genes. This technique excels in selecting features that maximise classification performance, even in complex datasets (**Figures 3C, D**). Random Forest utilises out-of-bag error estimation and Gini importance scores to identify 17 hallmark genes with diagnostic significance. Its robust ensemble learning approach ensures that important features are consistently identified, even when faced with noisy or high-dimensional data (**Figures 3E, F**). By synthesising the outcomes from all three algorithms using a Venn diagram, we identified ten diagnostic ERDEGs, which were consistently highlighted across the different approaches (**Figure 3G**). This integrated feature selection strategy ensures the robustness and reliability of the final gene set for HNSCC risk modelling.

### Clinical validation of diagnostic models

**Figure 4:**
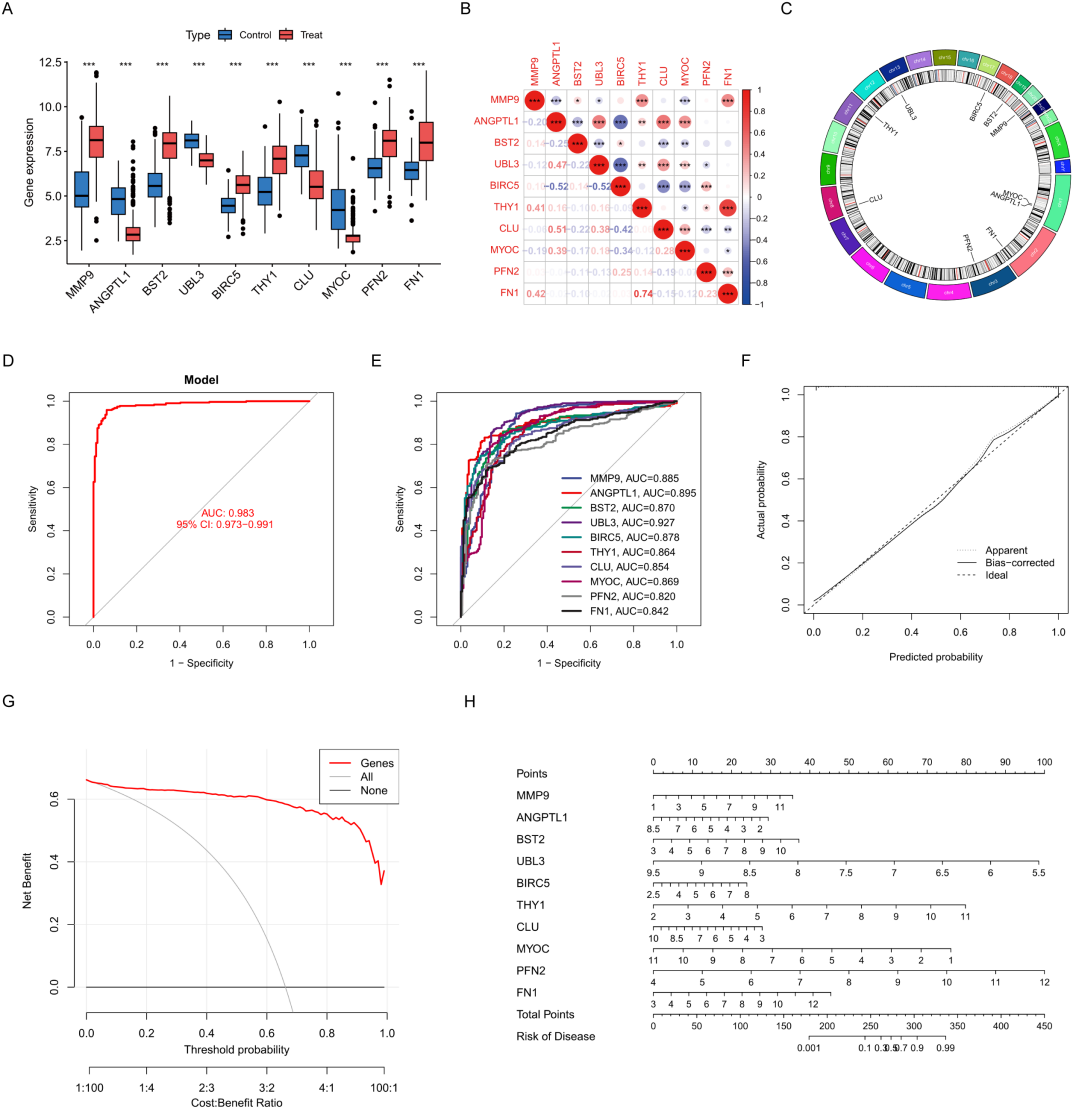
Construction and characterisation of characterisation genes. **(A)** Box line plot comparing gene expression in control and treated groups. **(B)** Correlation plots reveal expression relationships between genes. **(C)** Loop plots demonstrate the distribution and association of genes on chromosomes. **(D)** Model ROC plot to assess overall diagnostic performance. **(E)** ROC plot for each gene. **(F)** Calibration curve graph compares predicted probability with actual probability. **(G)** Decision curve plots measure the net benefit of clinical applications. **(H)** Column line graphs construct individualised risk prediction models. Data were showed as mean ± SD, *P < 0.05, **P < 0.01, ***P < 0.001.

Boxplot analysis demonstrated significant differential expression of critical genes between control and treatment groups (p < 0.001 for all comparisons). Genes such as matrix metallopeptidase 9 (MMP9), ANGPTL1, bone marrow stromal cell antigen 2 (BST2), ubiquitin-like 3 (UBL3), baculoviral IAP repeat containing 5 (BIRC5), Thy-1 cell surface antigen (THY1), clusterin (CLU), myocilin (MYOC), profilin 2 (PFN2), and fibronectin 1 (FN1) demonstrated distinct expression profiles, with MMP9 and FN1 exhibiting the most significant upregulation in the treatment group (**Figure 4A**). Correlation analysis revealed intricate relationships among the genes. FN1 highly correlated with THY1 (r = 0.74, p < 0.001). BIRC5 had inverse correlations with ANGPTL1 (r = -0.52) and UBL3 (r = -0.52). CLU demonstrated moderate co-expression with ANGPTL1 (r = 0.51) (**Figure 4B**). The Circos plot analysis delineated critical genes to particular chromosomal regions. CLU (chromosome 8) and THY1 (chromosome 11) are in regions that regulate the extracellular matrix. UBL3 on chromosome 13 and BIRC5 on chromosome 17 are located in regions associated with apoptosis (**Figure 4C**). To evaluate the diagnostic efficacy of pivotal genes identified by the LASSO risk model for HNSCC, logistic regression diagnostic models and column line plots were employed to demonstrate the impact of the expression of 10 selected ERDEGs on HNSCC. ROC curve analysis designated UBL3 as the most potent single-gene biomarker (AUC = 0.927, 95% CI: 0.901–0.953), surpassing other possibilities such as ANGPTL1 (AUC = 0.895) and MMP9 (AUC = 0.885) (**Figure 4E**). The multivariate model encompassing all genes attained remarkable diagnostic accuracy (AUC = 0.983, 95% CI: 0.973–0.991), greatly above that of individual markers (**Figure 4D**). To rigorously evaluate model generalizability, we performed independent validation using the TCGA-HNSCC dataset (n = 546), which was completely independent from all prior training and feature selection procedures. The diagnostic model achieved near-perfect discrimination with an AUC of 0.999 (95% CI: 0.996–1.000) (**Supplementary Figure 2A**). Individual biomarkers demonstrated robust predictive capacity, including BIRC5 (AUC = 0.962), MMP9 (AUC = 0.951), and ANGPTL1 (AUC = 0.889), with all 10 genes showing AUC > 0.75 (**Supplementary Figure 2B**). Calibration curves exhibited robust concordance between projected probabilities and actual outcomes (Brier score = 0.083), with negligible discrepancy between apparent and bias-corrected estimates (**Figure 4F**). The decision curve study validated clinical utility within 10–80% threshold probabilities, demonstrating enhanced net benefit relative to treat-all or treat-none approaches (**Figure 4G**). The nomogram assessed the contributions of individual genes to disease risk, with UBL3 (5.5–9.5 points) and FN1 (3–12 points) exhibiting the highest weightings. Total scores of 300 points or higher indicated a predicted risk exceeding 90%, facilitating accurate categorising of high-risk patients (**Figure 4H**).

### Immune microenvironment characterisation

**Figure 5:**
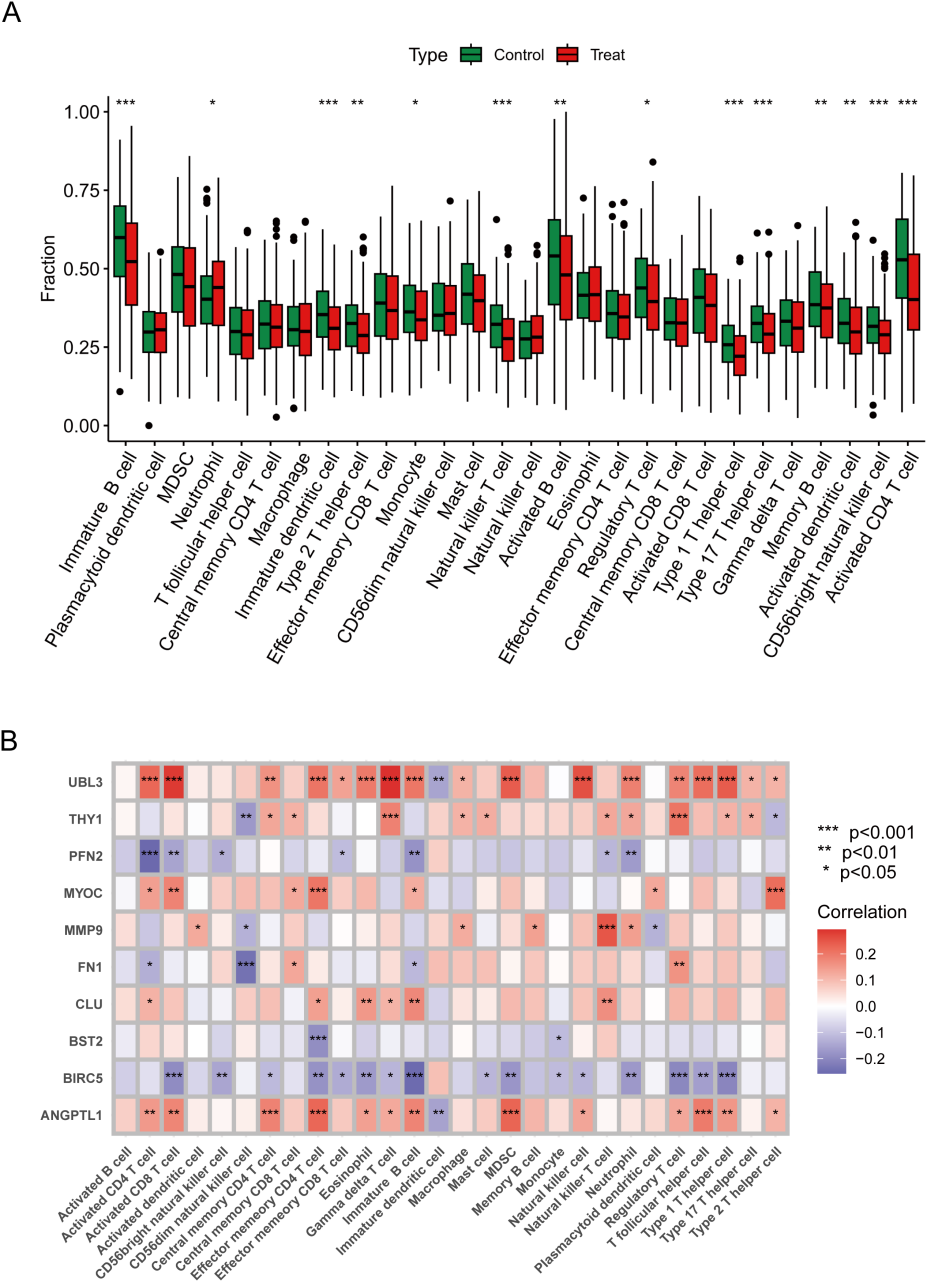
Immune properties of characterised genes. **(A)** Immune difference plot comparing the change in distribution of immune cells between control and treated groups. **(B)** Correlation heatmap demonstrating correlation and significant differences between genes and immune cells. Data were showed as mean ± SD, *P < 0.05, **P < 0.01, ***P < 0.001.

An examination of immune infiltration was conducted using the CIBERSORT method to investigate the link between immunoreactivity and HNSCC, revealing the infiltration of 28 immune cell types, with 14 kinds exhibiting significant differences between the treatment and control groups. Neutrophils were more prevalent in HNSCC, but Natural Killer T cells, Activated CD4 T cells, Activated B cells, and Memory B cells were more prevalent in the control group (**Figure 5A**). The Spearman analysis demonstrated a link between immune cells and ERDEGs, as illustrated in **Figure 5B**. UBL3 was prevalent in activated CD8 T cells, gamma delta T cells, myeloid-derived suppressor cells (MDSCs), and natural killer cells, exhibiting a favourable correlation with inflammation-related signalling pathways, potentially contributing significantly to the control of innate immunity. BIRC5 exhibits a strong negative correlation in Immature B cells, Activated CD8 T cells, and Regulatory T cells, suggesting that these innate immune cells are inhibited during T cell proliferation. ANGPTL1 is prominently expressed in effector memory CD4 T cells and myeloid-derived suppressor cells (MDSCs), potentially contributing to immunosuppression and the regulation of the tumour microenvironment. MYOC is significantly expressed in effector memory CD4 T cells and type 2 T helper cells, indicating its potential influence on antigen presentation functionality.

### Therapeutic target exploration

**Figure 6:**
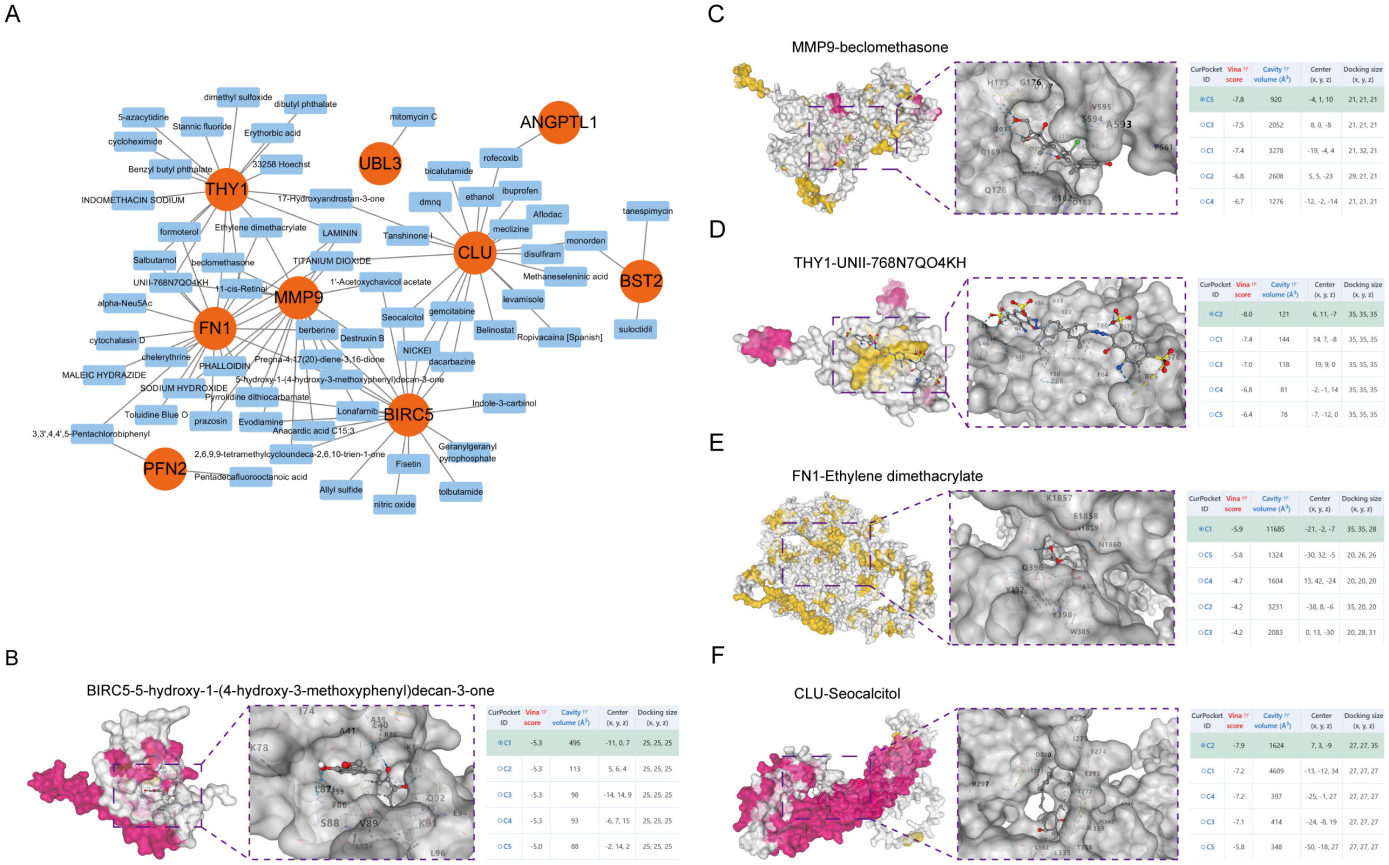
Molecular docking of small molecule drugs. **(A)** Drug Regulatory Networks. The 3D structure of molecular docking shows the results of molecular docking of BIRC5 with 5-hydroxy-1-(4-hydroxy-3-methoxyphenyl)decan-3-one **(B)**, MMP9 with beclomethasone **(C)**, THY1 with UNII-768N7QO4KH **(D)**, FN1 with Ethylene dimethacrylate **(E)**, and CLU with Seocalcitol **(F)**.

Small molecule medicines modulating hub gene expression were gathered from DSigDB on the Enrichr platform. The outcomes for prospective small molecules were produced using their P-values to signify the closeness between the small molecule and the gene. **Figure 6A** and **Supplementary Table 2** illustrate the prospective small molecule therapeutics for the hub genes. To clarify the binding activity between the hub gene proteins and their respective medications, additional molecular docking of the HNSCC-related hub genes (BIRC5, MMP9, THY1, FN1, CLU) and the initial five small-molecule medicines was conducted. Consequently, receptor-ligand docking outcomes were acquired utilising the identical methodology. In molecular docking, intermolecular forces, primarily hydrogen bonding, were considered. **Figures 6B–F** depicts the docking configuration of small molecule pharmaceuticals and proteins.

### Regulatory network analysis

**Figure 7:**
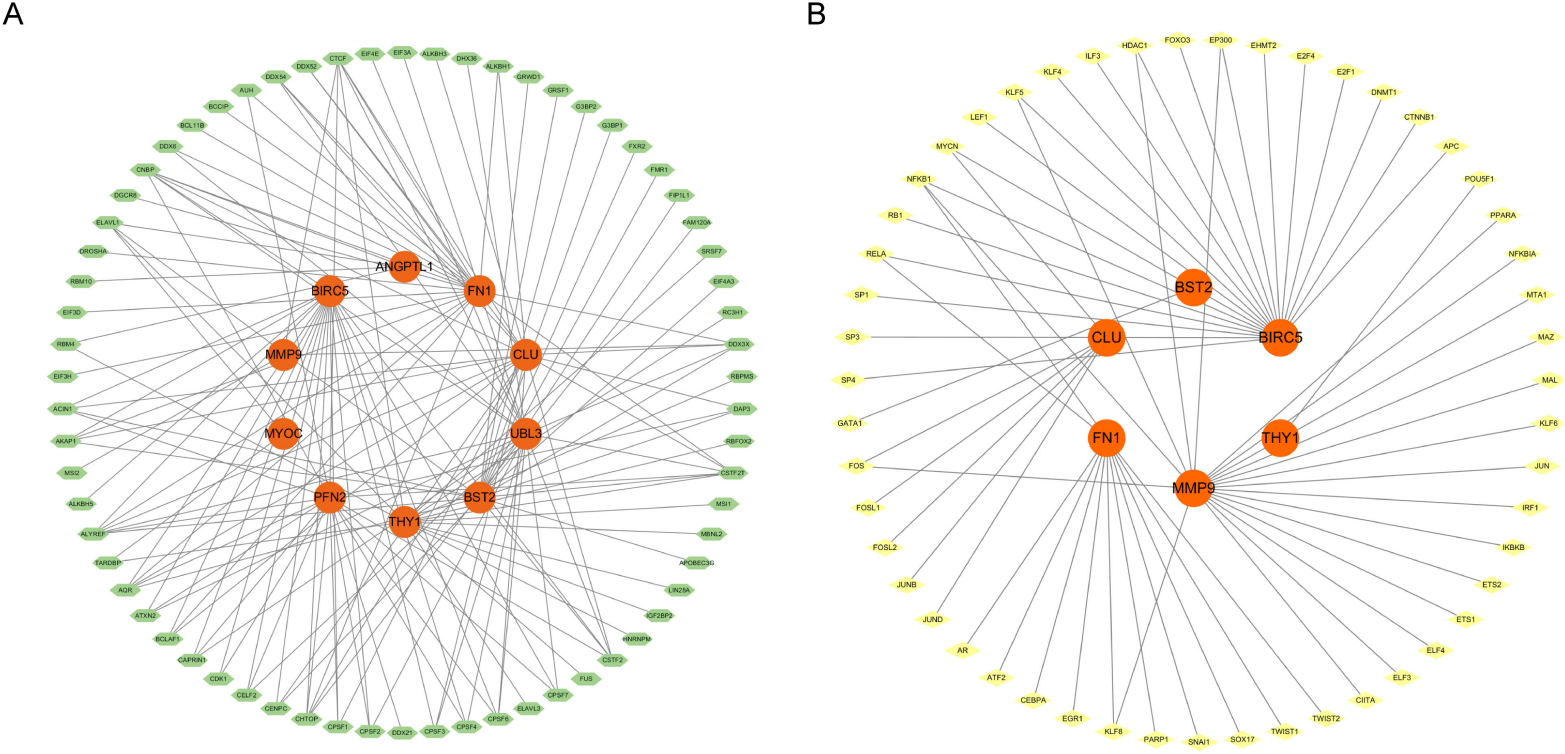
**(A)** RBP regulatory network diagram (RNA binding protein). **(B)** TF transcription factor regulatory network diagram.

This study established a regulatory network for RBPs, with green nodes denoting RBPs and orange nodes indicating target genes. Central to the network, genes, including BIRC5, FN1, CLU, MMP9, and UBL3, were co-regulated by various RBPs. BIRC5, an established anti-apoptotic gene integral to cell survival and carcinogenesis, is modulated by several RNA-binding proteins and may be intricately regulated at the post-transcriptional level. FN1 is an extracellular matrix protein essential for cell adhesion, migration, and tissue repair, and its interactions with several RBPs indicate a sophisticated regulatory mechanism at the RNA level (**Figure 7A**). This study also established a TF regulatory network, wherein yellow nodes denote TFs and orange nodes signify target genes. Central to the network, genes including MMP9, BIRC5, CLU, BST2, and THY1 were co-regulated by various transcription factors. MMP9, a gene integral to extracellular matrix disintegration and cancer spread, is modulated by many transcription factors and may be meticulously regulated throughout cellular migration and tissue remodelling. BIRC5, an anti-apoptotic gene crucial for cell survival and carcinogenesis, is regulated by many transcription factors, indicating its modulation by different signalling pathways at the transcriptional level (**Figure 7B**).

### ANGPTL1 inhibited HNSCC cell proliferation, migration, and invasion

**Figure 8:**
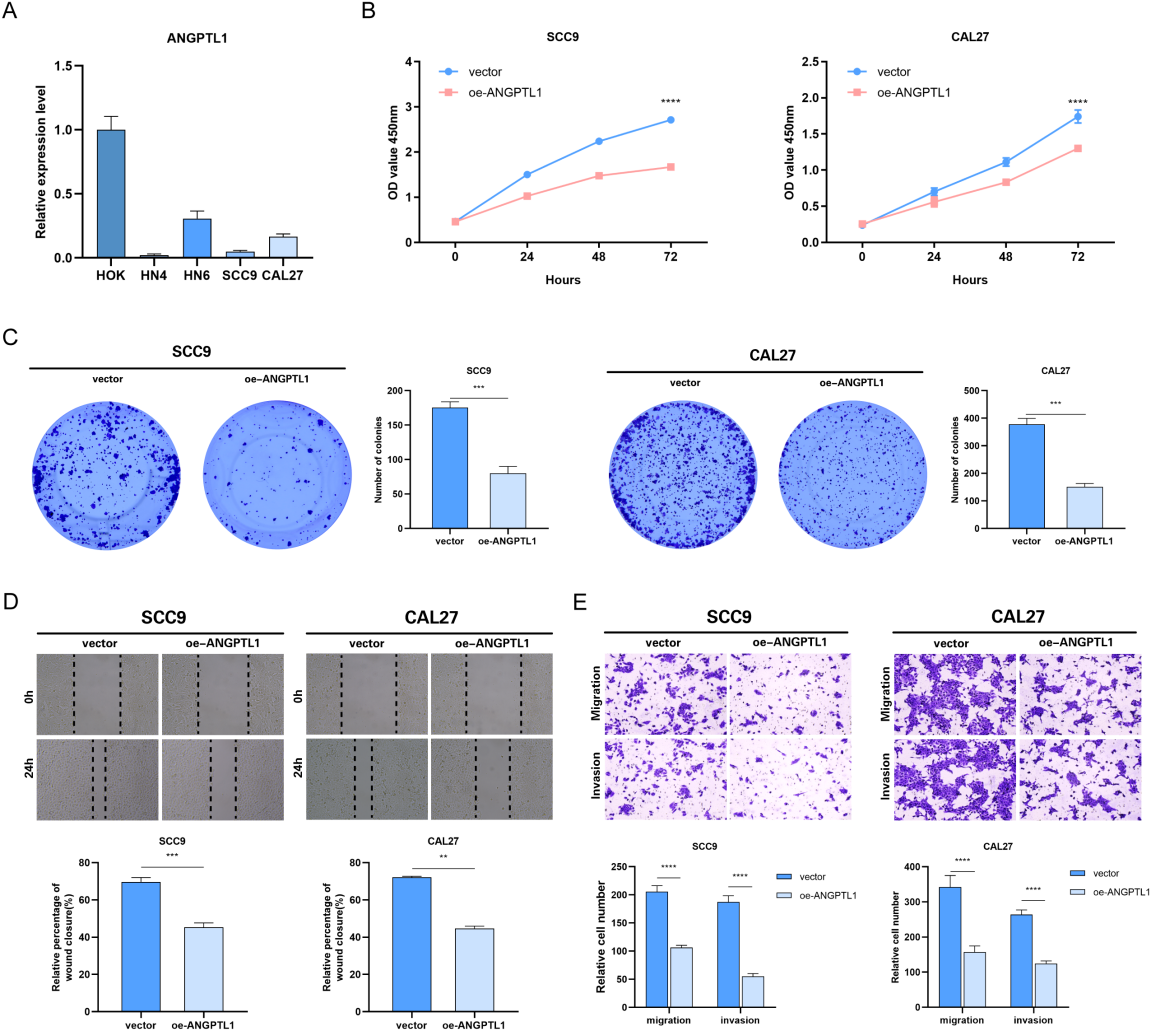
Effect of overexpression of ANGPTL1 on the functional phenotype of HNSCC cells. **(A)** Results of qRT-PCR assay of ANGPTL1 mRNA in various HNSCC cell lines. **(B)** CCK-8 proliferation assay of HNSCC cells affected by overexpression of ANGPTL1. **(C)** Clone formation assay to verify the effect of overexpression of ANGPTL1 on the proliferation of HNSCC cells. **(D)** The effect of overexpression of ANGPTL1 on the migration ability of HNSCC cells was verified by scratch assay. **(E)** Transwell assay was performed to verify the effect of overexpression of ANGPTL1 on the migration and invasion ability of HNSCC cells. Data were showed as mean ± SD, *P < 0.05, **P < 0.01, ***P < 0.001, ****P < 0.0001.

Using qRT-PCR to detect the differences in ANGPTL1 mRNA expression among different HNSCC cell lines, the ANGPTL1 mRNA expression levels in HNSCC cells were significantly lower than those in the HOK cell line (**Figure 8A**). HNSCC samples from the HPA database showed absent ANGPTL1 protein expression (staining intensity score = 1), while normal oral mucosa maintained moderate expression (score = 2) (**Supplementary Figure 3A**), and expression was further reduced in patients with TNM stage II-III (stage I/II vs stage III/IV: log2FC = 0.47, p = 0.0038) (**Supplementary Figure 3B**). Selected two head and neck squamous cell carcinoma cell lines, SCC9 and CAL27, with low ANGPTL1 expression as subjects for subsequent research. We created a model for overexpression of the ANGPTL1 gene and introduced vector-NC and oe-ANGPTL1 into HNSCC cells by transfection. The results of the CCK-8 experiment showed that the cell proliferation rate in the ANGPTL1 overexpression group of SCC9 and CAL27 cells was significantly lower than that in the control group cells (**Figure 8B**). The results of the colony formation assay showed that the cell cloning ability of the ANGPTL1 overexpression group in SCC9 and CAL27 cells was significantly inhibited (**Figure 8C**). To further verify the role of ANGPTL1 protein in the migration and invasion abilities of HNSCC cells, scratch assay results showed that in SCC9 and CAL27 cells, the cell migration ability in the ANGPTL1 overexpression group was significantly lower than that in the control group (**Figure 8D**). In the transwell experiment, after ANGPTL1 protein overexpression in HNSCC cells SCC9 and CAL27, a decrease in the transmembrane invasion ability of the cells was observed (**Figure 8E**).

## Discussion

This study utilised sophisticated machine learning methods to systematically discover exosome-related indicators in HNSCC, a cancer marked by significant morbidity and death. We found 10 ERDEGs with substantial diagnostic and prognostic potential by integrating multi-omics data and analysing the immunological microenvironment. UBL3 was identified as a strong single-gene biomarker with an AUC of 0.927, whereas a combined model utilising all 10 ERDEGs had outstanding diagnostic accuracy (AUC = 0.983). These findings underscore the effectiveness of machine learning in transforming intricate information into clinically applicable insights. The development of a nomogram facilitated accurate risk classification, with a total score beyond 300 points associated with over 90% disease risk, highlighting its effectiveness in individualised patient management. Genes such as THY1, FN1, and BIRC5 function as diagnostic markers and demonstrate significant correlations with immune cell infiltration and tumour growth, indicating their dual involvement in disease identification and therapeutic intervention.

MMP9 facilitates tumour invasion and metastasis through the degradation of the extracellular matrix (16). In HNSCC, elevated MMP9 expression was substantially correlated with lymph node metastases and unfavourable prognosis (17, 18). Exosomes transport MMP9 to distant tissues, altering the microenvironment to establish a pre-metastatic niche and increasing the invasiveness of HNSCC (19). ANGPTL1 functions as an anti-angiogenic agent and a tumour suppressor (20, 21). ANGPTL1 is downregulated in several malignancies, and multiple studies have evidenced its inhibitory function in tumour growth and metastasis (22, 23). Exosomal ANGPTL1 reprograms Kupffer cells and reduces their MMP9 expression, averting hepatic vascular leakage and impeding colorectal cancer liver metastases (24). BST2 participates in immunological modulation and viral suppression (25, 26). In HNSCC, the overexpression of BST2 may enhance tumour cell survival by activating the AKT/ERK1/2 pathway and is linked to immune evasion (27). UBL3 modulates the ubiquitin cascade process (28). Recently, UBL3 was identified as a post-translational modification that facilitates protein sorting into tiny extracellular vesicles (29). BIRC5 is an anti-apoptotic protein that significantly influences cell proliferation, differentiation, migration, and invasion (30–33); its elevated expression in HNSCC is associated with treatment resistance and unfavourable prognosis (34, 35). THY1 participates in the regulation of cell adhesion and migration (36, 37). Research indicates that THY1 on the surface of extracellular vesicles (EVs) or the receptor cell surface interacts with corresponding integrins to facilitate the binding, uptake, and distribution of EV contents (38). The function of THY1 in intracellular vesicles remains unclear; nevertheless, it has been identified in non-follicular vesicles and neuronal synaptic vesicles (39). CLU functions as a molecular chaperone that participates in stress response and the regulation of apoptosis (40, 41). In oral cancer cells, CLU overexpression enhances the activation of the AMPK/Akt/mTOR-mediated autophagy pathway, hence promoting cell survival (42). MYOC is mainly linked to glaucoma and has received limited research attention in the context of cancer (43). There is insufficient evidence to establish a direct involvement in HNSCC development or exosome function; nonetheless, it may indirectly influence tumour behaviour through the modulation of ECM hardness, warranting additional investigation. PFN2 modulates the reorganisation of the actin cytoskeleton (44). In HNSCC, PFN2 enhances tumour invasiveness via epithelial-mesenchymal transition (EMT) (45). PFN2 promotes tumour angiogenesis within the tumour microenvironment via cancer-derived exosomes (46). FN1 is an essential extracellular matrix element that facilitates tumour cell adhesion, motility, and metastasis (47–49). In HNSCC, elevated FN1 expression correlates with MDSC infiltration and an immunosuppressive microenvironment (50). Exosomal FN1 can stimulate fibroblasts through integrin signalling, facilitating pro-carcinogenic ECM remodelling and enhancing metastasis (51).

Functional enrichment analysis indicated that ERDEGs are primarily associated with pathways essential to HNSCC pathogenesis, including TNF signalling, IL-17 signalling, and ECM-receptor interactions. These pathways are pivotal to inflammation, immune evasion, and metastasis. FN1, a crucial extracellular matrix protein, promotes tumour cell adherence and migration, and its noted association with heightened infiltration of Activated CD8 T cells and MDSCs (52, 53). BIRC5, an anti-apoptotic gene, exhibited an inverse correlation with regulatory T cells, suggesting its role in inhibiting anti-tumour immune responses (54). *In vitro* experiments provided experimental support that ANGPTL1 plays an anti-cancer role, inhibiting the proliferation, migration, and invasion of HNSCC cells. These mechanistic findings highlight the diverse functions of exosome-related genes in influencing tumour biology via intracellular signalling and extracellular communication within the tumour microenvironment.

Examining immune infiltration patterns in HNSCC tissues indicated a tumour-promoting environment characterised by increased neutrophils and reduced natural killer T cells. ERDEGs such as UBL3 and ANGPTL1 displayed substantial connections with immunosuppressive cell types, including MDSCs, suggesting their involvement in immune evasion. Notably, UBL3 was associated with Activated CD8 T cells and pro-inflammatory pathways, highlighting its contradictory involvement in immune activation and tumour growth. MMP9 and FN1 were linked to extracellular matrix remodelling, a process essential for forming metastatic niches (55–57). Based on these findings, drug sensitivity estimates and molecular docking revealed prospective therapeutic drugs targeting essential ERDEGs. BIRC5 showed affinity for anti-mitotic agents such as berberine, aligning with its function in cellular survival (58), whereas THY1 and FN1 were anticipated to engage with immune checkpoint inhibitors, reinforcing their promise in combinatorial therapy designed to augment anti-tumour immunity.

Notwithstanding these gains, some limits merit attention. During the development of the model, we employed cross-validation as well as multiple feature selection methods to minimise the risk of overfitting. However, despite this, overfitting is still a concern, especially in the case of high-dimensional datasets. To reduce the risk of overfitting, we suggest that future studies should conduct further external validation and consider applying more stringent regularisation techniques to improve the reliability and generalisation of the model. Although *in vitro* investigations offered preliminary insights into ANGPTL1’s functional significance, extensive *in vivo* studies are necessary to clarify the molecular contributions of other ERDEGs, including their role in immune regulation. Translational initiatives might also benefit from experimental confirmation of anticipated medication interactions using patient-derived models, such as organoids or xenografts. Furthermore, future research should isolate tumour-specific exosomes to directly correlate ERDEGs expression with exosomal cargo and functional outcomes, thereby refining our understanding of exosome-mediated intercellular communication in HNSCC progression.

## Conclusion

In conclusion, this work employed a machine learning methodology to uncover dependable exosome-related biomarkers for HNSCC. We conducted an extensive bioinformatics analysis to thoroughly investigate exosome-associated genes’ expression patterns and functional roles in HNSCC, emphasising their significant contribution to tumour growth and immune modulation. A molecular docking study indicated distinct interactions between exosome-associated proteins and pharmacological targets. These findings highlight the significance of exosomes in cancer biology and offer new avenues for future translational research focused on enhancing the early diagnosis of HNSCC, personalised therapy approaches, and patient prognosis.

## Data Availability

The original contributions presented in the study are included in the article/**Supplementary Material**. Further inquiries can be directed to the corresponding authors.
