# Supplementary material for: Machine learning-driven identification of exosome- related biomarkers in head and neck squamous cell carcinoma
Source: Front Immunol. 2025 May 22;16:1590331. doi: 10.3389/fimmu.2025.1590331 (PMC12137257; doi:10.3389/fimmu.2025.1590331)
Supplement: Supplementary file 1 [file DataSheet1.docx]

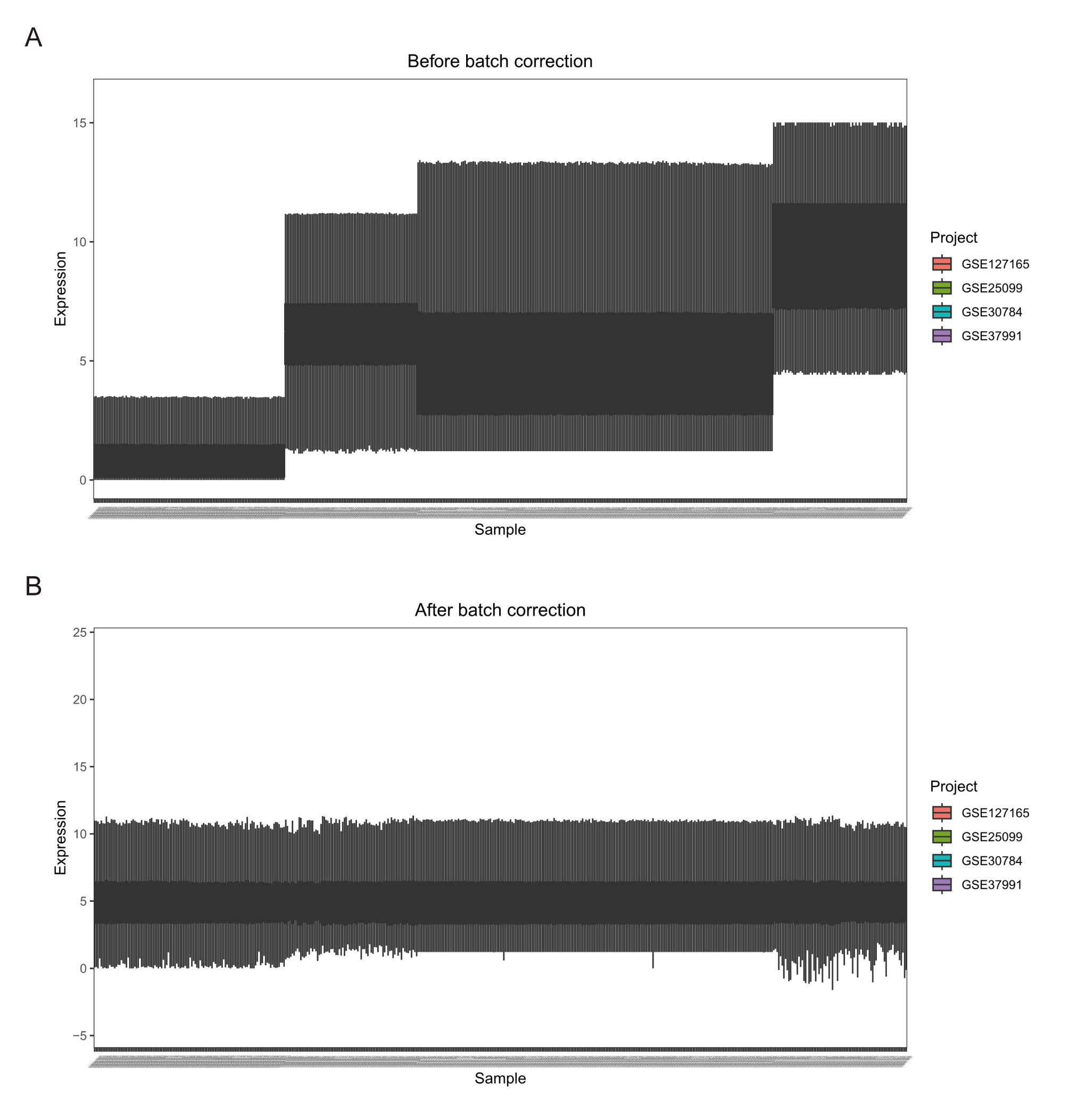


**Supplement Figure 1.** **(A)** There were significant between-sample differences and batch effects in the raw expression data before batch correction. **(B)** Normalization and batch correction resulted in a more focused data distribution and convergence of expression across samples.
