# Supplementary material for: Machine learning-driven identification of exosome- related biomarkers in head and neck squamous cell carcinoma
Source: Front Immunol. 2025 May 22;16:1590331. doi: 10.3389/fimmu.2025.1590331 (PMC12137257; doi:10.3389/fimmu.2025.1590331)
Supplement: Supplementary file 2 [file DataSheet2.docx]

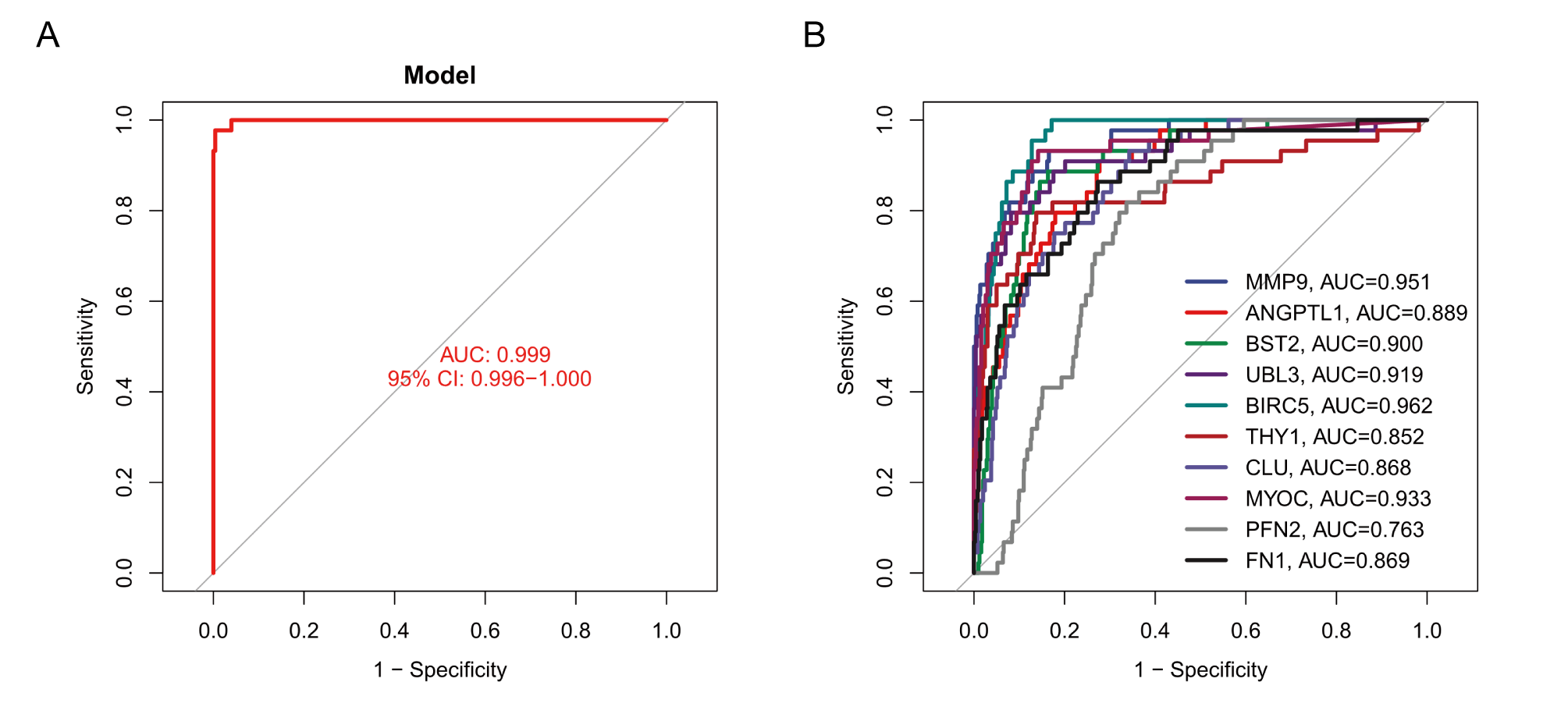


**Supplement Figure 2. (A)** This ROC curve shows that the predictive model has near-perfect diagnostic performance. **(B)** In the combined multigene ROC curve, the BIRC5 gene had the most prominent predictive efficacy (AUC=0.962), with all genes having AUC values > 0.75.
