# Supplementary material for: Machine learning-driven identification of exosome- related biomarkers in head and neck squamous cell carcinoma
Source: Front Immunol. 2025 May 22;16:1590331. doi: 10.3389/fimmu.2025.1590331 (PMC12137257; doi:10.3389/fimmu.2025.1590331)
Supplement: Supplementary file 3 [file DataSheet3.docx]

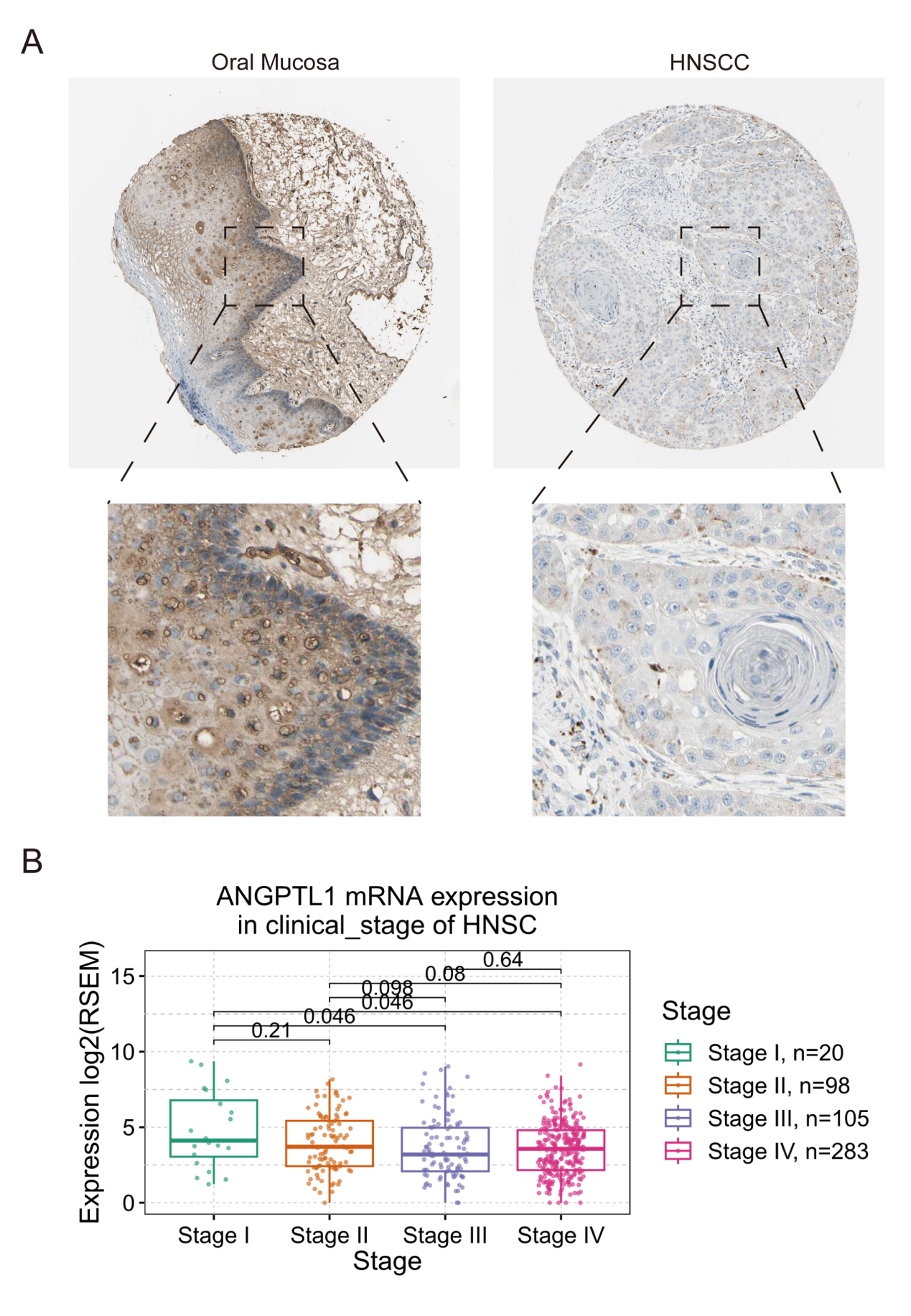


**Supplement Figure 3. (A)** Immunohistochemical staining of ANGPTL1 in normal oral tissues and HNSCC tissues from HPA. **(B)** Comparison of ANGPTL1 expression in different subgroups of clinical stage.
